# Creation and application of virtual patient cohorts of heart models

**DOI:** 10.1098/rsta.2019.0558

**Published:** 2020-05-25

**Authors:** S. A. Niederer, Y. Aboelkassem, C. D. Cantwell, C. Corrado, S. Coveney, E. M. Cherry, T. Delhaas, F. H. Fenton, A. V. Panfilov, P. Pathmanathan, G. Plank, M. Riabiz, C. H. Roney, R. W. dos Santos, L. Wang

**Affiliations:** 1 King’s College London, London, UK; 2 University of California San Diego, La Jolla, CA, USA; 3 Imperial College London, London, UK; 4 University of Sheffield, Sheffield, UK; 5 Georgia Institute of Technology, Atlanta, GA, USA; 6 Maastricht University, Maastricht, the Netherlands; 7 Ghent University, Gent, Belgium; 8 Center for Devices and Radiological Health, U.S. Food and Administration, Rockville, MD, USA; 9 Medical University of Graz, Graz, Austria; 10 Universidade Federal de Juiz de Fora, Juiz de Fora, Brazil; 11 Rochester Institute of Technology, La JollaRochester, NY, USA; 12 Laboratory of Computational Biology and Medicine, Ural Federal University, Ekaterinburg, Russia

**Keywords:** simulation, cardiac, virtual patient cohorts, digital twin

## Abstract

Patient-specific cardiac models are now being used to guide therapies. The increased use of patient-specific cardiac simulations in clinical care will give rise to the development of virtual cohorts of cardiac models. These cohorts will allow cardiac simulations to capture and quantify inter-patient variability. However, the development of virtual cohorts of cardiac models will require the transformation of cardiac modelling from small numbers of bespoke models to robust and rapid workflows that can create large numbers of models. In this review, we describe the state of the art in virtual cohorts of cardiac models, the process of creating virtual cohorts of cardiac models, and how to generate the individual cohort member models, followed by a discussion of the potential and future applications of virtual cohorts of cardiac models.

This article is part of the theme issue ‘Uncertainty quantification in cardiac and cardiovascular modelling and simulation’.

## 1. Introduction

Hindsight is a wonderful thing. If we know what will happen, it is easy to make the right decision. Biophysical patient-specific models strive to encode known physics and physiology within mathematical equations and to tune these models to represent individual patients. The aim is to use these *digital twins* to predict disease progression, better estimate risk and predict treatment response so that the outcome might be known before a decision is made. With sufficiently accurate predictions, the choice of the best treatment for a patient shifts from being based on the current or past condition of the patient to the future one. While conceptually simple, the practical reality of determining the equations, tuning the parameters to patient data and generating reliable predictions remains a significant engineering and mathematical challenge.

Overcoming such engineering and mathematical challenges has huge potential. Once a patient-specific model is created, it can then be re-used to design new treatments, evaluate inclusion criteria, simulate imaging or diagnostic signals, or test mechanistic hypotheses. In contrast to recent advances in statistical regression models—which are limited to cases where large datasets are already available—biophysical models are based on physical laws and known physiological systems and so have greater versatility in their predictions, mechanistic explanatory power and susceptibility to analysis.

There has been significant investment in the development of biophysical models. Cardiac modelling, in particular, has made major recent advances in moving patient-specific modelling into the clinic [1]. The move to human-scale simulations has driven the development of efficient and scalable code that is required for the simulation of large human hearts [2–4]. The ability to simulate human hearts increased the ability to tune models to clinical datasets, motivating the use of image- and signal-processing techniques to convert medical images into data that could be used to constrain the models. This work has culminated in the recent use of models to guide therapies in prospective studies of ventricular tachycardia ablation [5], atrial fibrillation ablation [6] and cardiac resynchronization therapy lead positioning [7].

This shift from developing research or proof-of-concept models to using models in clinical workflows requires a step change in speed and robustness in model creation [8]. A patient-specific model needs to be created from standard clinical data robustly and reliably, assessed and interrogated, all within a short time frame, often less than 24 h. As the cost, in time, of model creation decreases significantly, this enables the development of large virtual cohorts of models. These virtual cohorts will allow inter-patient variability to be captured in cardiac model simulations. These virtual cohorts will allow virtual trials (VTs), which will impact clinical care through therapy design and development, including patient selection and therapy guidance.

The development of virtual patient cohorts poses new opportunities for cardiac modelling. How best to move from a cottage industry, where each model is handcrafted, to an industrialized process where models are produced en masse with limited to no human intervention, is a challenge. This white paper discusses the current state of patient-specific cardiac models; the process of developing virtual patient cohorts; how we validate these models; how we quantify uncertainty at the level of the individual model and at the level of the virtual cohort; and finally potential and future applications of virtual cohorts.

## 2. Examples of virtual cohorts of cardiac models

Table 1 provides an overview of representative studies which used virtual cohorts of cardiac models. To date, these studies have included a relatively small number of individuals, between 5 and 100 virtual patients. In contrast, algorithmic studies may be applied to much larger clinical datasets; for example, Pennells *et al.* computed cardiovascular disease risk algorithms using data from 360 737 participants by pooling data across 86 prospective studies [25]. Constructing virtual patient computational modelling cohorts on such a scale is not currently possible because of the challenges associated with data availability, as well as the time and specialist methodology required to construct these detailed biophysical models.

**Table 1.** Patient-specific modelling studies: number of models and study goals.

reference	number of patients	goal of the study	type of model	strategy
[9]	35 samples from ex vivo RAA	atrial model calibration	0D	RVAC
[10]	4 CRT upgrade; 14 de novo implantation	predicting activation with CRT devices	BV	1:1
[11]	24 ICM	mechanisms for arrhythmia risk with CRT	LV	1:1
[12]	46 HF	building personalized models	BV + T	1:1
[13]	7 clinical cases with ICD	building personalized models	LV	1:1
[14]	7 PAF cases	building personalized models	LA	1:1
[15]	5 CT cases with torso	calculating a shock efficiency metric	BV + T	1:1
[5]	RS: 5 swine; 21 humans (5 with ICD) PS: 5 humans	guiding the ablation of infarct-related ventricular tachycardia	BV	1:1
[16]	4 PAF; 16 PsAF	simulating different ablation strategies	LA	1:1
[17]	12 PsAF	simulating ablation of inter-atrial connections	LA	1:1
[18]	7 PAF, 5 PsAF	simulating AF pre- and post ablation	LA	1:1
[19]	108 PsAF	simulating empirical versus computer-guided ablation	LA	1:1
[6]	4 PAF; 6 PsAF	computationally guided personalized ablation	BA	1:1
[20]	118 PsAF	computationally guided personalized ablation	LA	1:1
[21]	5 AF patients	stroke risk assessment in AF (CFD)	LA	1:1
[22]	70 (training)+60 (test)+3 (12 k samples)	shape uncertainty	LA	SID
[23,24]	5 PsAF	patient-specific modelling of atrial action potentials		1:1

CT, computed tomography; CRT, cardiac re-synchronization therapy; HF, heart failure; HCM, hypertrophic cardiomyopathy; ICM, ischaemic cardiomyopathy; ICD, implantable cardioverter defibrillator; RAA, right atrial appendage; PAF, paroxysmal atrial fibrillation; PsAF, persistent atrial fibrillation; PS, prospective study; RS, retrospective study; 0D, cell model; BV, bi-ventricular; LV, left ventricle; BV + T, bi-ventricular + thorax; LA, left atrium; BA, bi-atrial; 1:1 = 1:1 mapping virtual cohort; SID, sampling from inferred distributions; RVAC, random variation with acceptance criteria.

The studies in table 1 focused on aspects of model construction and calibration, on obtaining mechanistic understanding of a disease or on predicting response to a therapy. For example, Cedilnik *et al.* presented a modelling pipeline for constructing fast personalized ventricular models using an eikonal formulation [13], demonstrating their methodology on seven CT and electrophysiology datasets. Corrado *et al.* [14] presented a workflow to build personalized computational models from local multi-electrode catheter measurements and applied the technique to data from seven paroxysmal atrial fibrillation clinical cases. Kayvanpour *et al.* generated a larger cohort of models consisting of 46 heart failure patients, incorporating personalized anatomy, electrophysiology, biomechanics and haemodynamics [12]. As well as personalizing the cardiac anatomy, models may include a torso mesh from imaging data [15]. The effect of variability is also important; Muszkiewicz *et al.* investigated this by integrating cellular-level and ion channel recordings in human atrial models using right atrial appendage measurements from 35 patients [9].

Multiple studies have used computational models to test response to therapies, including ablation of atrial arrhythmias [16–19], ablation of ventricular arrhythmias [5], CRT optimization [10] and ventricular tachycardia risk assessment following CRT [11]. These studies vary in the degree of model complexity and personalization included and the size of the virtual cohort. For example, atrial ablation studies range from smaller studies, incorporating left and right atrial fibrosis distributions in bilayer models (*n*=12 [17]), or left atrial volumetric models including transmural fibrosis (*n*=12 [18]), to large studies using left atrial shell models for which only the anatomy was personalized (*n*=108 patients [19]).

## 3. Approaches to generating a virtual cohort of cardiac models

The aim of virtual cohorts of cardiac models is to account for inter-patient variability in simulation studies. A virtual cohort consists of multiple members, where each member of the cohort has a distinct parameter set. The variation in parameter sets and anatomy between members aims to represent the variability in the true patient population.

### (a) Strategies for generating virtual cohorts

**Figure 1.**
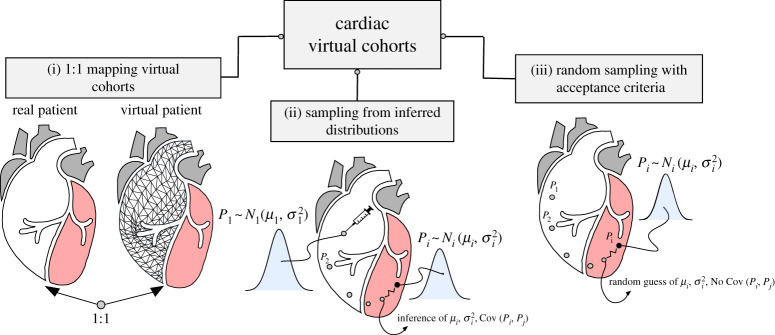
Schematic of the strategies for obtaining a virtual cohort, based on biophysical models. (Online version in colour.)

The parameter set for each member of the virtual cohort can be obtained in three ways: first, by having each member of the virtual cohort represent a specific patient from a real-world cohort (*1:1 mapping*); second, by generating a parameter set from inferred parameter distributions (*sampling from inferred distributions*); and, third, by completely randomly generating parameters and testing if these result in physiologically plausible models (*random variation with acceptance criteria*). Figure 1 gives a schematic summary, while the methods are detailed below.

#### (i) 1:1 mapping virtual cohorts

The development of 1:1 mapping virtual cohorts incrementally builds on current techniques for creating models of a specific patient’s heart: it repeats this process multiple times in order to generate a number of specific models that, in turn, form the virtual patient cohort. While superficially simple, the process of repeating the patient creation workflow on new patients is often subject to subtle variations or the effect of artefacts in clinical data. Manual steps quickly become bottlenecks, data structures need to be standardized and cost functions that were tailored to the first case need to be generalized for all cases. In addition, the first case is always built on the best and most complete data. Moving to multiple cases exposes the challenges of obtaining multiple high-quality datasets from specific patients. This makes data collection for the generation of large virtual cohorts time-consuming and expensive. Nonetheless, the availability of data is essential also for other strategies aiming at the construction of virtual cohorts, and a number of research groups are working on this area, addressing each of challenges mentioned above [1,24,26–29].

#### (ii) Sampling from inferred distributions

In cases where a 1:1 mapping virtual cohort can be created for a representative subset of a population, it is possible to also infer the parameter variability and co-variability. This allows one to model the parameters as following a statistical distribution. By sampling from these distributions, new parameter sets can be generated representing new virtual members of the patient population. The statistical distribution of the parameters can be assumed to be of known form—for example, Gaussian. The task, in this case, reduces to using the data to estimate the hyper-parameters of the distribution—corresponding to the mean vector and the variance–covariance matrix in the Gaussian example. Alternatively, if there are no principled reasons to assume a known form for the parameter distribution, this can be inferred applying either Bayesian statistical methods [30] such as Markov chain Monte Carlo (MCMC) [31], or frequentist techniques such as bootstrapping [32], to individual or cohort data. The resulting parameter distribution is a discrete (or sample) approximation of the underlying distribution. In both Bayesian and frequentist approaches, parameter sets for members of the virtual cohort can then be generated by drawing from the inferred distributions that have the property of representing the variance and covariance structure of the parameters emerging from the data. While this approach allows estimation of the effects of population variance, there are no guarantees that the generated cohort members will be physiologically plausible and, ideally, each member model should be evaluated to ensure a physiological plausibility.

#### (iii) Random variation with acceptance criteria

In situations where the variance and co-variance of the model parameters is unknown, and sufficient measurements to infer a distribution of parameters are not available, then it is possible to generate large numbers of parameter sets by randomly varying parameters to generate new members of a virtual cohort. Variations of this approach were first taken by [33,34]. It is possible for each proposed member of the virtual cohort to be evaluated against population measurements and only models that fall within these physiological bounds to be included in the virtual cohort, as performed in [35] and subsequent papers. This is a robust method, which is simple to implement, and allows virtual cohorts to be built when limited data or summary statistics or processing time are available, and provides measures of how parameter variability might affect results, by producing high-dimensional plausible regions in the parameter space. Differences between virtual cohorts can then be evaluated by the degree of overlap in model predictions generated by the population. However, despite the formal similarity to the MCMC accept–reject procedure, the method fails to account for co-variation in model parameters and measured phenotypes; the parameter sets that are generated are possible but there is no guarantee that they occur physiologically; as more variables are added to define acceptance criteria, it becomes less likely that any individual is near the mean of the multivariate population distribution; finally, while parameter sets are generated these are not samples from probability distributions, so standard statistical tests for differences between populations should not be applied.

### (b) Synthetic versus patient-derived members of the virtual patient cohort

The strategies for model development can be separated into development of *synthetic virtual patients*, where virtual patients are generated by sampling from distributions (whether inferred or guessed and not-rejected) and *patient-derived virtual patients*, generated using the 1:1 mapping approach, in which case the virtual patients correspond to actual real people. While the data cost for each patient is high in the 1:1 mapping approach, this does provide some guarantee that each virtual patient’s heart will operate within some physiologically plausible space. In the case where only a limited number of models can be made, due to time or data restrictions, using a 1:1 mapping approach has the potential for patient-specific bias. The bias, if any, can potentially be estimated by comparing emergent model phenomena with population statistics from larger clinical trials or from population databases. On the other hand, the generation of synthetic virtual patients allows speculative studies to evaluate what could potentially happen in extreme edge cases. This could be useful when trying to identify rare events or edge patient cases, who may not be represented in available patient cohorts. However, this approach runs the risk of creating non-physiological or implausible models that can skew the results for the virtual cohort, especially in the case where parameters are randomly guessed. However, once 1:1 mapping cohorts of sufficient size have been generated, these provide better bounds and parameter distribution estimates for constraining synthetic virtual patient approaches.

## 4. Constructing, constraining and validating virtual cohort models

**Figure 2.**
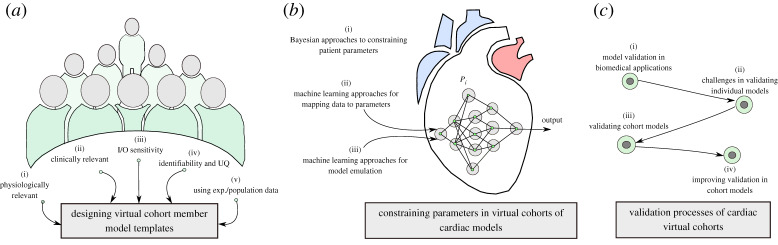
Process of creating a virtual cohort. (*a*) Defining a template model structure for the members of the cohort, (*b*) constraining the parameters for the members of the virtual cohort and, (*c*) validating the models representing specific individual patients and the virtual cohort. (Online version in colour.)

Creating a virtual cohort requires the development of a template model for representing each member of the cohort. The template needs to be carefully designed to be able to capture patient variability, physiology, diseases and treatments of interest. In cases where the model is tied to clinical data for specific patients, the model complexity needs to reflect the available clinical data and the time and resources available to create the model. In virtual cohort strategies, where models need to be tuned to represent all or a subset of specific patients, the model parameters must be inferred using nonlinear optimization, statistical or machine learning approaches. The constrained model is subsequently exposed to a validation step, in order to assess its generalization properties for predictions tasks. These three steps are depicted graphically in figure 2.

### (a) Designing virtual cohort template model templates

A virtual patient cohort is made of virtual members who all share a common model structure. This means that variability is encoded in the anatomy, physiological parameters and boundary condition parameters, as opposed to differences in model structure. As with previous cardiac biophysical models, while the initial aim of a virtual patient cohort may be specific, if virtual cohorts are to be reused outside of their original application, the model template will need to be designed to be generic, reusable and easy to manipulate, allowing simulations to be run and analysed at scale. Ideally, the model template will be combined with a definition of the physiological envelope within which the member and virtual cohort models have been validated to ensure appropriate use and reliable predictions.

#### (i) Physiologically relevant virtual cohort member model templates

The model template must encode physiologically relevant mechanisms for the virtual cohort application. The level of physiological detail in a model template needs to balance complexity versus the ability to constrain model parameters.

Increased complexity is motivated by the lack of a clear set of known important physiologically relevant mechanisms and the desire to make a general virtual cohort that can be applied to multiple applications. However, complex biophysical models often rely on representative or population parameters and this may miss important patient-specific physiology aspects. By contrast, the use of simple models is motivated by the intent of constraining most (if not all) the model parameters to be patient specific, as well as the inability to precisely constrain all the parameters of a complicated model by the available data, and the need to contain simulation costs [23,36].

As a minimum, when tested, simulations of individual patients within the virtual cohort should be able to reproduce the corresponding clinical measurements from that patient to within a specified tolerance. The model parameters and predicted phenotypes should be prescribed into the mathematical model structure to ensure that the model captures fundamental physiology and that parameters are globally identifiable from the available data [37].

#### (ii) Clinically tractable virtual cohort member model templates

As described, cohorts of virtual patients can be created in two ways. First, synthetically by sampling parameter distributions, as part of an offline focused project to create a virtual patient cohort. Second, from a cohort of patient-derived virtual patients created as part of a clinical trial or routine clinical care. When models need to be made at scale, or as part of the clinical workflow, the time taken to create each model becomes critical. As models in the near future are likely to be created as an adjunct to standard clinical practice, the data used to constrain model parameters and validate model predictions need to reflect available clinical data. Adhering to these two steps will allow virtual patient cohorts to fit directly into clinical applications, in which parameters are inferred by matching patient physiological dynamics [38].

#### (iii) Parameter and predicted phenotype sensitivity of virtual cohort member model templates

Model sensitivity is another important challenge in designing the virtual cohort member template model. Sensitivity is defined as the rate of change in simulated model predictions or outputs in response to changes in model parameters. For an example, see [39]. In biophysical cardiac models, the mapping from patient data to simulation predictions can be separated into two steps. First, the dependence of model parameters on input data can be determined. This sensitivity can be used for informing how values are measured [40,41], for example choosing between echocardiography, MRI or CT to measure cardiac mechanics to achieve a desired precision in inferred model parameters. Second, the dependence of the model outputs on model parameters can be determined. Parameters to which the model output of interest is relatively insensitive may not need to be personalized, whereas parameters to which the output is highly sensitive may need to be precisely personalized for each patient. Examples of sensitivity analysis applied to cardiovascular diseases, where model output measures are used to reflect clinical goals, can be found in [42,43].

#### (iv) Identifiability and uncertainty quantification of virtual cohort member model templates

In cases where models are based on patient-specific clinical data, the template model parameters, or a defined subset, should be derived from the available data. In the case where a single deterministic parameter set is used to reflect the physiology of each member of the virtual cohort, the model parameters should be uniquely identified for each specific patient. However, there is growing application of uncertainty quantification (UQ) to patient-specific models. Within this framework parameters are defined by distributions as opposed to a single value. The distribution of a parameter for a given patient represents the uncertainty of the true parameter value for that patient. An advantage of the UQ approach is that it provides a natural framework for predictions: these are simply generated from multiple instances of the same patient, with parameters randomly sampled from the distributions in each simulation. In both cases, the creation of a virtual cohort based on the inferred parameters identifies a population level distribution.

#### (v) Using experimental or population data in virtual cohort member model templates

The need to capture physiologically relevant mechanisms, create identifiable models and develop models rapidly and robustly has led to pragmatic modelling choices. For example, in tissue electrophysiology simulations, detailed representative biophysical cell models are used that are not tuned to the individual patient [5,6]. However, these models capture the complex interplay of cellular electrophysiology and calcium dynamics that are important in arrhythmia simulations and allow qualitatively relevant predictions to be made that have been successfully used to inform patient treatment. Setting a non-personalized parameter to a single fixed value is often the only practical option due to computational resource constraints. However, it would be more correct to view non-personalized parameters as uncertain and specify them using probability distributions, ideally representing population variability in that parameter conditional on known information about the patient (e.g. on sex or age). This approach is currently infeasible for whole-heart patient-specific models due to both computational cost and lack of information on population (and sub-population) variability for the wide assortment of functional parameters in cardiac models. However, the increased availability of population databases, for example the UK biobank [44] and, in the future, the availability of virtual cohorts of cardiac models, will provide population-based priors for informing some non-personalized model parameters in individuals. This will allow more sophisticated models to be made that are informed by a combination of population and patient-specific data, and that fits more and more the UQ paradigm.

### (b) Constraining parameters in virtual cohorts of cardiac models

With the rapid growth of clinical and consumer sensor technology, which make the gathering of large individual and population patient data readily available, there is an increasing need for accurate big data analytics to construct and constrain virtual cohorts of cardiac models. The tools are supplied by emerging fields such as big data informatics and machine learning [45,46], used to inform model parameters from large datasets. For example, modern deep data techniques can be used to analyse and constrain larger numbers of model parameters, which increases understanding of how patient-data could be used and shared. On the other hand, machine learning—especially its probabilistic version [47]—provides efficient approaches for clustering, dimensionality reduction and constraining models. These methods are thus becoming ubiquitous in the way virtual cohort models and data are used in medicine.

#### (i) Nonlinear optimization and Bayesian approaches

The most intuitive approach for constraining the parameters of a cardiac physiological model is to minimize a cost function that represents the distance between the model evaluated in a given set of parameters and the data. A number of cost functions can be considered, that, for example, weight differently each data point, or that add a penalty of some sort on the magnitude of the parameter vector. The task is thus to find the values of the parameters by means of iterative nonlinear optimization algorithms [48–50], where the nonlinearity is intrinsic in the way the parameters enter the cardiac model. The main drawback of this approach lies in the multimodality of the cost function: this is both a computational challenge for numerical algorithms, which need to have the ability to learn multiple minima, and it poses interpretability issues in a case where there is not a unique global minimum.

A natural solution is offered by Bayesian inference, that forms a large part of the UQ methods mentioned above. In fully Bayesian approaches, a prior belief on the parameters (a distribution with typically population-based parameters) is modified to a posterior belief by means of observed patient data, whose information is modelled through the chosen likelihood function. Inference corresponds, thus, to the ability of sampling from the posterior distribution of the parameters: this is, for example, the key step for obtaining predictions from the model, as explained above, but another typical task is using the samples to approximate integrals of interest (for example, the posterior mean). MCMC methods are the main class of algorithms for full posterior sampling with batch data, based on a principled accept–reject scheme. When the data are seen as arriving sequentially in time or space, and one wants to capture the intrinsic stochasticity of the sequential data, the corresponding class of Bayesian learning algorithms is provided by sequential Monte Carlo methods [51]. These generalize the Kalman filter to non-Gaussian data and nonlinear models; we refer to [52] for an electrophysiology example, aimed at constraining the Mitchell and Schaeffer model. A number of challenges arise in these seemingly easy procedures, primarily related to the difficulty of designing efficient proposal distributions, especially when the parameter of interest is high-dimensional and the posterior is multimodal. Computational solutions have been provided by means of (i) approximation of the posterior, as in variational Bayes approaches [53], approximate Bayesian computation, [54,55], posterior tempering [56] and dimension reduction [57]; (ii) direct exploitation of the geometry of the posterior [58]; (iii) state-space augmentation to ease the inference [59]. Finally, restarting MCMC methods multiple times from different initial points allows one to identify at least some of the different posterior modes, and to design a sampler with improved capacity of exploring high probability regions.

#### (ii) History matching approaches

When the variance–covariance structure of the parameters is not of strict interest, or it too costly to obtain, history matching (HM) provides an approach to constrain the parameters to *plausible* regions. The model is evaluated for a large space-filling design of parameter values (e.g. latin hypercube), which are accepted or rejected based on an implausibility criterion. As an example, in [60], HM is used to constrain a subset of the parameters of the Courtemanche and the Maleckar cell models. The main difference between the MCMC and the HM acceptance criteria is that the former, if successfully converged, guarantees that all the accepted parameter values are (correlated) samples from the posterior distribution, although to assess the convergence of MCMC chains remains a challenging task in itself [61]. The latter simply identifies regions of the parameter space that are consistent with the measured data, trying to account for uncertainty in both observations and predictions, but without guarantees. To tackle the computational burden of evaluating a very large number of samples, MCMC and HM often rely on a model surrogate or emulator, as described in a later section.

#### (iii) Machine learning approaches for mapping data to parameters

Data-driven machine learning has the advantage of extracting complex relationships from large amounts of data, giving a model which later can be executed efficiently for time-sensitive workflows. This approach will complement virtual cohorts of cardiac models that are biologically and physiologically sophisticated but face challenges in assimilating data from different sources in a timely fashion. There are thus many opportunities for integrating machine learning methods with virtual cohorts of cardiac models.

Machine learning models can be trained to learn a direct mapping between model parameters and outputs generated by virtual cohorts of cardiac models, which can later be used to allow a fast calibration or UQ of virtual cohorts of cardiac models when given clinical data. In [62], a polynomial regression model was trained to predict myocardial electrical diffusion from simulated ECG data, which was then used for fast calibration of a cardiac electrophysiology model from clinical ECG data. Similar ideas were explored in [63,64] for personalizing cardiac electrophysiology models from higher density surface ECG data. In [65], linear regression models and decision trees were learned to map input geometrical features to simulated haemodynamic features, which were then used for quantifying the uncertainty in haemodynamic outputs as a result of geometric uncertainty. These approaches provide an attractive time-effective alternative to calibrating or quantifying the uncertainty in virtual cohorts of cardiac models in clinical workflows, in contrast to traditional optimization and statistical inference which are typically computationally expensive. The challenge regarding the multi-modality or non-identifiability of the parameter given available data, however, still remains, highlighting the importance of UQ to characterize the probabilistic distribution in the model parameters even in machine learning approaches [66]. An additional challenge arises from how well the machine learning relationship trained on simulation data may generalize to clinical data, and this approach was suggested as a preliminary step prior to more refined model personalization in [62,67].

#### (iv) Machine learning approaches for model emulation

With the rapid growth in their modelling capacity, data-driven deep learning models may also have a role in directly approximating the simulation model for the purpose of accelerating data assimilation in virtual cohort models that are otherwise computationally prohibitive to realize. Similar to earlier surrogate/emulator models such as polynomial chaos and Gaussian process, the fundamental idea is to learn to approximate the simulation-based solutions and then use these computationally efficient surrogates in later tasks such as data assimilation [68,69], which traditionally consists of the optimal integration of typically sparse real-world olbservations to improve model estimates such as forecasts or state reconstructions [70]. This is rather appealing for enabling data assimilation of virtual cohort models at scale, although several challenges remain to be addressed. To build the training database from simulation remains time-consuming, and it is not clear how exhaustive the simulation needs to be in order for the machine learning surrogate to be able to mimic the simulation model over a wide range of parameter values [71,72].

### (c) Validation of virtual cohorts

Validation is the process of assessing whether a model is suitably representative of the physical process it seeks to represent and therefore whether predictions from the model are sufficiently close to those of the real system. It should be noted that there is no such thing as a *validated* model, but rather a body of evidence that the model produces results which are consistent with the physical system being modelled in a specified regime or parameter space. While a model may generate accurate predictions within one region of parameter space, it may not necessarily extend to producing reliable, or even physiologically plausible, results outside of that region [73]. Our confidence in a specific model output should, therefore, reflect its position relative to the regime in which validation has been undertaken. Furthermore, models will often predict variables that can not be or were not measured directly, for example stress in cardiac mechanics models. These variables can be of interest for understanding mechanisms underpinning emergent observations. There will be less confidence in these model predictions that can not be compared against experimental data. However, confidence in the model prediction can be gained if the model is physics based and is validated across a wide range of conditions that alter the unmeasured model output.

#### (i) Challenges in validating individual models

Cardiac models are often highly complex, frequently spanning multiple scales, from tissue properties to individual ion channels. Each of these sub-components includes assumptions and parameters for which evidence supporting their representation of reality should be sought. Few parameters correspond to directly observable quantities, requiring inference procedures in order to incorporate these observations into the model. These may use simple approaches (such as linear regression), or necessitate the use of more sophisticated statistical models to tease out the relevant associations. For example, tissue conductivity in homogenized models cannot be measured experimentally/clinically. Statistically, models can be used to associate this quantity with an observable, such as conduction velocity. In calibrating a cardiac model to an individual, observations are often only acquired in a small region of the parameter space (e.g. during pacing or sinus rhythm) or at low spatio-temporal resolution, depending on time, practical and ethical constraints. The process of validation often requires evaluating the model multiple times; the complex nature of whole-heart cardiac models can make this computationally expensive and time-consuming, adding further challenge to the validation process.

#### (ii) Validating cohort models

These issues are further compounded when looking to validate cohort models. Independent of the approach used to generate the cohort model, we would like to validate it against real patient observations, or real cohorts of patients. The method used to generate the virtual cohort strongly impacts the ability to perform validation. For example, virtual patients generated using the 1:1 mapping method correspond to actual patients, from which other data can be obtained for validation. Synthetic virtual patients, on the other hand, cannot be validated in the same way, as there is not a corresponding real patient to compare against. Cohorts of either type can be validated in a statistical manner by comparing cohort-level statistics against population-level statistics. Owing to the significant challenges in performing this validation, evidence supporting the validity of biophysical models in the existing literature tends to be sporadic and ad hoc. At the cellular scale, efforts to validate action potential models are generally quite prevalent. While verification of computational implementations of tissue-scale modelling has been proposed [74,75], limited validation against actual patient data is found in many whole-heart modelling studies [14]. Many cohort studies cite evidence supporting their validity from previous studies, which in turn cite earlier studies which ultimately provide limited actual evidence in themselves.

#### (iii) Improving validation in cohort models

To improve validation, each study should be able to reference a body of primary evidence (rather than earlier studies using the model) supporting the components of the model being used from earlier work, and include evidence that the model as a whole is representative of the population it aims to represent. This may include some cohort-level validation steps taken within the study, but also explicitly citing validation data for sub-components used, such as assessments of tissue-scale and action potential models, and their calibration methods, for the individual simulations within the cohort and for the regimes under consideration in the study. This procedure should be used in helping to establish the validity of results, and consequently the strength of conclusions drawn during the peer-review process. This process would be made more effective through the pursuit of specific (multiple, independent) validation studies, either published through traditional journals or as white papers on pre-print servers, which includes the raw data used. As part of this validation process, quantification and propagation of uncertainties in sub-components must inevitably play a role. Substantial work has already been done in the area of UQ for action potential models [76,77], but this needs to be propagated through to whole-organ models and cohort models [22]. Stating that a given outcome is the *most likely* outcome, given the available data, is a much stronger statement than a given outcome is *plausible*. Achieving all this, and being transparent about the extent to which the constituent components are themselves validated, will provide greater strength to cohort studies and document a clear and unambiguous provenance of validation evidence to support their use.

## 5. Potential and future applications of virtual cohorts of patient models

The creation of virtual cohorts of cardiac models are a relatively new innovation in cardiac modelling. These ideas have been proposed and adopted in other fields of computational modelling [78–80], including cardiovascular [81,82] and thymus modelling [83] as well as modelling of insulin and glucose [84,85], pacing lead design [85] and immunomodulation [86]. Currently, as described above, cohorts of cardiac models are being developed for specific applications to answer clinical or scientific questions. However, as the number of virtual cohorts developed and made publicly available increases, so too will the applications of these models.

### (a) Trial outcome prediction/proof of concept

One potential use of virtual cohorts is to simulate a clinical trial in advance of investing in the actual clinical trial. Assuming the simulations are expected to reliably predict the clinical endpoint of interest (difficult to ensure in practice), the advantages are clear: millions of dollars could be saved if the VT prevents a failing trial going ahead. For example, in [87], the authors used a VT to retrospectively re-create the results of the Rhythm ID Goes Head-To-Head (RIGHT) trial, which compared performance of two ICD devices. The conclusion of the RIGHT trial was the opposite of what had been hypothesized at the time; the virtual result reproduced this result.

### (b) Responder identification

Related to prediction of trial outcomes is the use of virtual cohorts to identify individuals who are likely, or not likely, to respond to the therapy, for example by identifying sub-populations within the intended patient population for which the proposed therapy is not likely to be effective. Studies using virtual cohorts could be used to derive better inclusion–exclusion criteria for the real trial, to reduce the size of the trial while maintaining adequate power. This could, depending on the effect size, ultimately be the difference between trial success or failure.

### (c) Trial augmentation and reduction

It has been proposed that some clinical trials could be augmented with a corresponding VT. Results from the VT, if they agree with the real-world trial, could be used to end the real-world trial earlier than initially planned. The medical devices community, via a collaboration facilitated by the medical devices innovation consortium (MDIC), has recently developed a Bayesian statistical framework for the formal integration of VT data and a real-world trial. The basic idea is a VT is performed in parallel with the real trial, and VT results are weighted according to the extent that they match the real-world trial. Specifically, the number of virtual patients used is controlled by a discount function which uses the similarity between modelled and observed data. This is a powerful approach because it reduces the (pre-use) validation burden for the computational models; results from the VT will essentially be discarded if they fail to match the real-world trial. The approach is described in [88].

### (d) Simulate situations for which clinical trials would be unethical

Virtual cohorts are already used in some applications to provide data for regulatory submissions, where performing a clinical trial would be impossible. Most notably, computational modelling has been used to evaluate safety of metallic implantable medical devices when the patient is exposed to the radiofrequency electromagnetic radiation during MRI. Implanted devices may heat and cause thermal tissue damage during MRI. Performing clinical trials to study whether heating remains within safe limits presents too great a risk to patients. Therefore, electromagnetic computational simulations are routinely used to predict potential MR heating for new implantable devices. A range of virtual patients—in this case, detailed whole-body anatomical models with electromagnetic material properties for each tissue—have been developed for these purposes. The virtual population [89] is a set of virtual patients, covering a range of ages, BMIs and both sexes. Some members were generated from segmentation of data from real subjects, others through morphing (synthetic virtual patients). Simulation studies using the virtual population have been used to establish RF safety in regulatory submissions for scores of devices [90].

### (e) Training machine learning algorithms using virtual cohorts

Virtual cohorts can be combined with machine learning approaches to generalize knowledge gained in virtual cohorts to future patient groups. In this setting, virtual cohorts have the ability to generate the high volumes of data required by machine learning and deep learning models that are otherwise difficult, expensive or impossible to obtain in experimental/clinical environments. This provides a way of generating low-cost high-volume synthetic data to initialize machine and deep learning models, which can then be retrained on potentially smaller but more relevant datasets. At the same time, machine learning models can mine from data generated by virtual cohort models and convert it to actionable knowledge for decision-making in the future. Several challenges exist in this process. How to address the discrepancy between the virtual cohort models and the reality? How to introduce sufficient variations in the virtual cohorts such that the derived machine learning models can generalize well when applied? Rapid advances in related machine learning concepts such as transfer learning and domain adaptation are likely to help us resolve these challenges, as demonstrated in recent work [64,91].

### (f) Virtual trials replacing clinical trials

The above applications use virtual cohorts to improve trial design (including whether to perform a clinical trial at all), reduce the size of a trial, provide evidence when a clinical trial is not possible or develop new algorithms. Ultimately, the holy grail for virtual cohorts is to replace clinical trials that are currently used to establish safety and efficacy/effectiveness of medical products. The current exponentially increasing cost of bringing medical products to market demonstrates the urgent need for cheaper, more efficient (but equally reliable) methods; computational modelling provides one potential solution [92]. Should virtual cohorts become successful in the above applications, it may become feasible for some clinical trials to be replaced by VTs. However, the current limited use of virtual cohorts in the above applications demonstrates that we remain far from this ambitious goal. The numerous challenges described throughout this paper, both related to cohort development and validation, will need to be robustly addressed, as will other challenges, for example, the social challenges of ensuring public confidence in such approaches.

## 6. What is needed to extend the application of virtual cohorts of cardiac models

### (a) Creating member template models with a hierarchy of complexity

Virtual cohorts of cardiac models should include member template models that contain models of different complexity and possibly should have modular structure. The top-end models should be based on the best available imaging of the object and should include detailed multiscale representation of all involved physiological properties with proper description from (sub)cellular to the whole organ level. However, we should also have a hierarchy of models for the same patients with lower spatial accuracy and a more generic description of physiological properties. The type of model used should be dictated by its specific application. The creation of top-end models still remains challenging due to problems with proper data collection and also due to insufficient understanding of some underlying physiological processes.

### (b) Omics driven models

In view of the huge amount of data obtained using genomics and proteomics data, it would be useful to connect the cardiac model parameters to the characteristics measured from omics. One of the most straightforward ways to do this would be to use widely available mRNA expression data and tune conductivities for the corresponding ion channels [93]. Another interesting approach was recently proposed in [94], which uses a novel methodology of Cap-Analysis of Gene Expression to tune model parameters to patient-specific data. In addition, as we obtain more and more data on cell regulatory systems, it would be good to add such data to electrophysiological models as was done, e.g., by Tan *et al.* [95] for cardiomyocyte mechano-signalling. It would be good to extend similar approaches to other regulatory systems.

### (c) Modelling tissue substrate

The fine structure of cardiac tissue is very complex and heterogeneous, and its features have a significant impact on heart function. Usually, the micro-anatomical organization of cardiac myocytes is modelled by spatial fields of cardiac fibre-sheet. The conduction system, in particular the Purkinje network (PN), is another structure that critically influences cardiac function [96]. Ex vivo imaging provides valuable information that has motivated the development of different rule-based models for both fibre-sheet fields and the PN. Unfortunately, these rule-based models are not patient-specific, and recent studies revealed that uncertainties on fibre-sheet fields [97,98] and on the PN models [99] considerably impact the results of cardiac simulations. Fibrosis is present in many cardiovascular diseases, and it is known to participate as both trigger and substrate of arrhythmias. Many proof-of-concept studies have shown the importance of the cell-scale and intricate pattern of fibrosis [100,101]. However, today’s non-invasive techniques only provide coarse-grained information about its location and shape. In the absence of fine-grained characterization of fibrosis, the amount of uncertainty significantly increases and challenges patient-specific modelling. In [29], to evaluate the pro-arrhythmic nature of a fibrotic region, the construction of a patient-specific model involved a collection of 500 biventricular models, each one representing a different but yet possible cell-scale pattern of the patient’s fibrotic region. Fortunately, emerging imaging techniques are expected to contribute with patient-specific information about the fine structure of cardiac tissue [102].

### (d) Publicly accessible virtual cohorts

The creation of patient-specific cardiac models requires access to patient data, access to tools to process the data and access to the compute resource required to run simulations to fit the model to the data. These all represent barriers to research groups developing patient-specific models, using virtual-patient cohorts or creating software to process and analyse simulation outputs. Recent interactive tools exploiting the computational power of relatively low-cost graphics cards [24] are designed to increase accessibility and can form the basis of a virtual-patient workflow. While repositories have been created for sharing patient data on public databases, for many groups this is not possible due to the use of historic data, data policies or question on data ownership. However, fully anonymized computational models of patients hearts that contain no clinical data are a lower barrier to sharing publicly. The publishing and sharing of virtual cohorts of cardiac models—analogous to the successful approach adopted in cardiac cell modelling—will accelerate the development and adoption of virtual cohorts of cardiac models.

## 7. Discussion and conclusion

The development of detailed biophysical virtual patient cohorts of cardiac models is an area of great potential but with many technical challenges. Interacting with industry and regulators will be important for the translation of virtual cohorts of patients into industrial and clinical tools. Modelling applications, including physiologically based pharmacokinetic models, provide an exemplar process for how to develop and validate models for use in clinical applications. Similarly, the use of models by industry in low regulatory early phases of device, drug or product development will provide a real-world context for developing and applying virtual patient cohorts. The increased complexity and computational cost in moving from a single patient to multiple patient modelling studies will require the creation of shared community resources. Improved access to simulation software, virtual cohorts of patients and model personalization workflows will facilitate the development and adoption of this modelling approach, and also reduce the number of projects that are forced to re-invent the wheel.

Virtual cohorts of cardiac models provide a low-cost tool for quantifying the impact of patient variability on physiology, pathophysiology and treatments. The ability to perform low-cost simulations over a meaningful representation of a patient population is an important step in the translation of computational models of the heart into industrial and clinical applications.

## Data accessibility

This article has no additional data.
